# Cyberbullying and Children and Young People's Mental Health: A Systematic Map of Systematic Reviews

**DOI:** 10.1089/cyber.2019.0370

**Published:** 2020-02-05

**Authors:** Irene Kwan, Kelly Dickson, Michelle Richardson, Wendy MacDowall, Helen Burchett, Claire Stansfield, Ginny Brunton, Katy Sutcliffe, James Thomas

**Affiliations:** ^1^ Evidence for Policy and Practice Information and Coordinating Centre (EPPI-Centre), Department of Social Science, Institute of Education, University College London, London, United Kingdom.; ^2^ Department of Public Health and Policy, London School of Hygiene and Tropical Medicine, London, United Kingdom.

**Keywords:** cyberbullying, children and adolescence, mental health effects, psychosocial well-being, systematic map

## Abstract

Cyberbullying is associated with considerable negative mental and psychosocial consequences in children and young people, making it a serious public health concern. To review the highest level of available evidence, a systematic mapping review was conducted to identify systematic reviews that investigated the relationship between cyberbullying and mental and psychological outcomes in young people. Topic-relevant bibliographic databases and online resources were searched to identify reviews published since 2007. Data were extracted using a coding tool developed for this study. Methodological quality of included reviews was assessed using AMSTAR criteria. Nineteen systematic reviews satisfied the inclusion criteria and they reported a strong negative association between cyberbullying and mental health outcomes in young people. Meta-analysis was performed in 11 reviews and narrative synthesis in 8 reviews. Data were derived from predominantly cross-sectional studies and a clear causal relationship between cyberbullying and mental outcomes cannot be established. Two-third of the included reviews were classified to be of low or unclear quality, due to the lack of quality assessment of the primary studies included in individual reviews. This systematic map consolidates available evidence at review level and confirms the existing gaps in longitudinal and qualitative evidence synthesis. Closer examination of the moderating factors influencing cyberbullying behaviors in future research can advance our understanding and inform the development of tailored programs of intervention to mitigate the negative impact of this phenomenon.

## Introduction

Internet-enabled electronic devices occupy a central part of the lives of many people, in particular, children and young people (CYP); from the use of computers and smartphones for school work and gaming to connecting with friends through social media.^1,2^ Since the introduction of the *iPhone* in 2007 and *android* in 2008, the technical functionality of screen-based devices has become more mobile and interactive, and so have their pervasiveness and use, leading to a rise in ownership of electronic devices by CYP from as young as three years of age.^3,4^ The near-universal Internet usage among CYP is highlighted by recent statistics. For example, in the United Kingdom, 99 percent of 12–15-year olds are now online.^4^ In the United States, 88 percent of teens have access to a desktop or laptop computer,^5^ 95 percent have access to various platforms through smartphones, and 45 percent say they are online “almost constantly.”^6^

Despite the benefits and opportunities afforded by Internet-enabled mobile technologies, there have been concerns about the growing rate of harmful online activities involving deliberate malice and harassment against CYP, such as cyberbullying. Social media platforms are very popular among teens and cyberbullying is reported to be most widespread on social media.^7,8^ Snapchat and Instagram have now overtaken Facebook in its popularity among young teens.^9^ National media coverage of teenage self-harm and suicides linked to cyberbullying has raised its political profile.^10,11^ Amid grave concerns shared by educators, health care professionals, parents, and CYP about online violence and internet safety, the U.K. government published the Digital Charter in 2018^12^ to set new rules and norms for the online world and launched an inquiry into the impact of social media and screen use on young people's mental health and well-being.^13,14^ This called for a duty of care on all social media companies in the form of a statutory code of conduct and transparent reporting.^15^ It is anticipated that new legislative measures will be implemented to ensure that internet platforms remove harmful content and prioritize the protection of users, especially children, young people, and vulnerable adults.

There is currently no consensus for what constitutes cyberbullying in the literature, with the use of a variety of related terms such as “cyber-aggression,” “internet harassment,” “online bullying,” and “electronic bullying,” making it difficult for researchers to accurately understand and distinguish the nature of cyberbullying from other forms of digital conflict and cruelty, such as online harassment and sexual harassment.^16,17^ In addition, cyberbullying has not been established as a causal precursor to satisfy the diagnostic criteria for mental health disorders such as post-traumatic stress disorder in DSM-V or ICD-10.^18,19^ However, most definitions of cyberbullying are modeled on the more widely accepted definition of traditional bullying,^16^ defined as acts of aggression that are repeated over time and that involve a power imbalance between the perpetrator and his or her targets.^20^ There seems to be some degree of similarity between traditional bullying and cyberbullying^21^ as both are reliably correlated,^22,23^ with cyberbullying being a continuation of traditional bullying executed through digital means.^24–26^ However, cyberbullying differs from traditional bullying as it involves a more extreme invasion of personal space, compounded by the potential anonymity provided to the perpetrator and the ability to harass regardless of the time of day,^27,28^ or the victim's whereabouts. It intrudes into spaces that have previously been regarded as safe and personal, such as the private environment of the home.^27,29^

There are various definitions of cyberbullying used in research.^8,25,28–33^ To unite the inconsistent definitions in literature, Tokunaga^34^ proposed the following:

Cyberbullying is any behavior performed through electronic or digital media by individuals or groups that repeatedly communicates hostile or aggressive messages intended to inflict harm or discomfort on others. (p. 278)

There is considerable variation in the reported prevalence of cyberbullying victimization among CYP, ranging from 4 to 72 percent,^25,33^ depending on the definition used, the age of the population studied, the tools of measurements used, and the research methodology employed. On average, 20–40 percent of CYP has experienced cyberbullying victimization at least once in their lives.^8,22,24,27,29,31,35–38^ Evidence suggests that cyberbullying can have a negative impact on CYP's mental and psychological health,^22,34^ and is strongly associated with depression, low self-esteem,^39^ and suicidal ideation.^40^

The increased research interest in this topic is reflected by a growing body of recent literature. To date, there has been no comprehensive review identifying, appraising, and summarizing existing evidence at review level on the relationship between cyberbullying and CYP's mental health and psychosocial well-being. This review was conducted in 2018 as part of a larger descriptive overview of existing review literature examining the relationship between screen-based activities and CYP's mental health and psychosocial well-being,^41^ commissioned by the Department of Health and Social Care UK. We aimed to consolidate existing knowledge by systematically mapping and reviewing evidence at review level on the mental health impacts of cyberbullying to inform decision making for educators, health care providers, and policy makers. Unlike a systematic review, a systematic map does not produce a meta-synthesis of findings, but rather an account of what evidence has been synthesized.^42^ This is beneficial for informing future research efforts by identifying research gaps and avoiding duplication of effort if the current evidence base is sufficient to inform policy and practice decision making.^43,44^ To meet our aim, we identified systematic reviews, described their key characteristics, and assessed their methodological quality. Our research questions were threefold:

- What is the nature and extent of systematic review literature on cyberbullying and CYP's mental health and psychosocial well-being?
- What is the quality of systematic review literature on cyberbullying and CYP's mental health and psychosocial well-being?
- What are the gaps in the systematic review literature evidence base and priorities for new evidence synthesis and primary research?

## Methods

This mapping review adheres to the Preferred Reporting Items for Systematic Reviews and Meta Analyses (PRISMA) guidance.^45^ Where necessary, the PRISMA guidance has been adapted to accommodate the systematic map approach taken.

### Systematic search strategy

Searches of 12 bibliographic databases that contain research literature on mental health, health care, social science, and education were carried out in August 2018: ASSIA (ProQuest), CINAHL PLUS (EBSCO), ERIC (EBSCO), EMBASE (OVID), Emerging Sources Citation Index (Web of Science), IBSS (ProQuest), MEDLINE (OVID), PsycINFO (OVID), Scopus, Social Policy and Practice (OVID), Sociological Abstracts (Proquest), and Social Science Citation Index (Web of Science). We also searched six other online resources: BASE, Epistemonikos, Google, Google Scholar, Schools Health Education Unit website, and the U.K. Safer Internet Centre website. Systematic reviews were also identified from title and abstract screening of a concurrent review undertaken at the EPPI-Centre.^46^

The search strategy was developed and implemented by an information specialist in collaboration with the lead reviewer. The search comprised three concepts that needed to be present in each of the study citations: (1) children, young people, or young adults; (2) cyberbullying; and (3) systematic review. Synonyms and alternative words for each of these concepts were used to search titles, abstracts, keywords, and controlled vocabulary fields of the databases to try to capture a wide range of systematic reviews. Journal fields were searched for the population concept. Other terms used for cyberbullying included cyber-victim, cyber-victimization, and where any of the terms, bullying, victim, victimization, harassment, aggression, and abuse occurred within two words of any of the terms cyber, internet, online, web, and website. The database searches were limited to citations published since 2007 in the English language, including gray literature. The search was undertaken as part of a broader search strategy that was developed to identify systematic reviews of social media, internet use, screen time on mental health, well-being, and risk-taking behavior.^41^ All citations retrieved from the entire search were assessed to determine their focus on cyberbullying. An example of the search history for PsycINFO is available from the authors on request.

### Eligibility criteria

To be included in the map, reviews needed to meet the following:

- Date: Be published in or after 2007
- Topic: Investigate the relationship between cyberbullying and mental health and/or psychosocial well-being
- Population: CYP younger than 25 years, as defined by WHO^47^
- Study design: Be a systematic review
- Language: Be published in English

### Data extraction

A coding tool was developed to extract information from the included systematic reviews to describe their key characteristics. For each review, we coded information about its aims, its scope based on the eligibility criteria applied, the search strategy employed, the number of databases searched and articles included, the study designs of the included studies, and the type of outcomes reported. An example of the coding tool is available in Supplementary Table S1.

Outcomes were coded according to whether they were answering a review question on the “associations” between cyberbullying and mental health or psychosocial outcomes, or answering a question on the longitudinal risk factors (precursors) or consequences of cyberbullying and mental health or psychosocial outcomes. It was deemed important to capture the reviews that had synthesized studies whereby cyberbullying (as an independent variable) was examined as a predictor of mental health or psychosocial outcomes, or as an outcome (dependent variable), along with mental health or psychosocial outcomes of an intervention study. A detailed description of the characteristics of the included reviews is available in Supplementary Table S2.

### Critical appraisal

We assessed the risk of bias of included reviews using the AMSTAR 2 criteria,^48^ which we modified and adapted to accommodate nonintervention studies. Details of the criteria are available in Supplementary Table S3. We categorized each review as having low, unclear, or high risk of bias for each AMSTAR domain using a framework shown in Supplementary Table S4.

### Data management and quality assurance

We piloted the eligibility criteria and coding tool by comparing decisions in groups of two reviewers using the systematic review software, EPPI-Reviewer 4.0.^49^ Citations identified by our searches were initially screened on titles and abstracts. Full reports from potentially eligible citations were then obtained and screened. At each stage of dealing with citations for the review (screening titles and abstracts and screening full reports), an initial sample of citations was double screened by reviewers independently and differences resolved by discussion. If agreement was adequate (e.g., 90 percent), the remaining citations were screened by a single reviewer alone. Where differences arose, they were resolved by seeking guidance from a third reviewer.

## Results

**FIG. 1.**
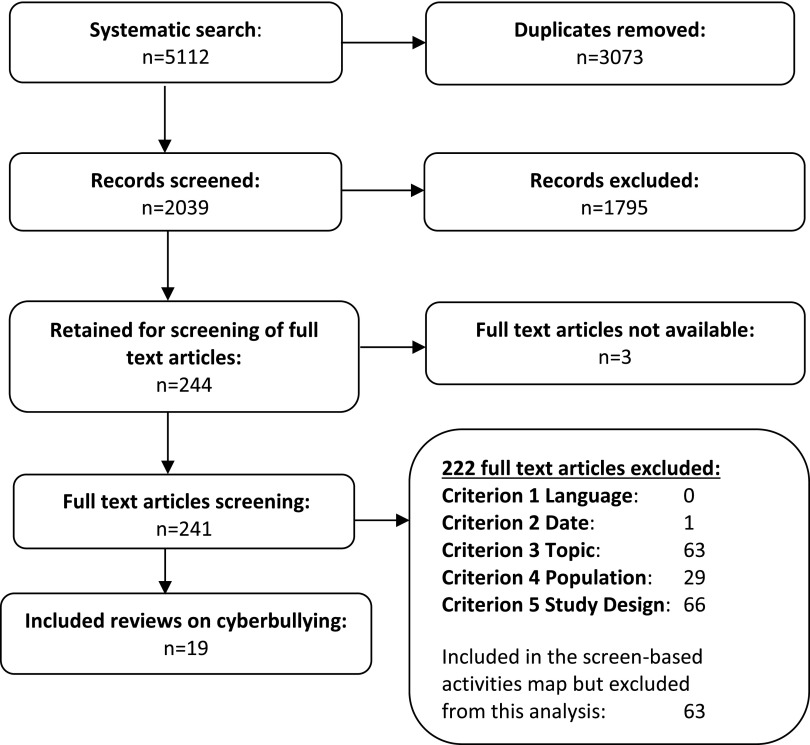
Flow of studies through the review.

We identified 19 systematic reviews^23,34,50–66^ (Fig. 1), which included a total of 832 primary studies. These investigated the relationship between cyberbullying and CYP's mental health and psychosocial outcomes. None of the reviews was restricted to longitudinal study designs only, and none conducted a qualitative evidence synthesis of CYP's views about cyberbullying.

### Characteristics of the included reviews

The date of publication ranged from 2010 to 2018 with the majority of reviews published in 2014 or later (*n* = 17). Most did not restrict studies by geographical location (*n* = 15), while four reviews only included studies conducted in high-income countries or countries which belong to the Organisation for Economic Co-operation and Development (OECD) group. Of the 19 reviews, 18 included primary studies of CYP populations only and 1 review involved participants of all ages. One review focused specifically on lesbian, gay, bisexual, transsexual, and questioning (LGBTQ) youth and one focused on known victims of bullying.

The dates searched among the primary studies ranged from 1910 to 2018. Five reviews did not state how far back they searched and two reviews stated that they searched from database inception. Four reviews specified that there was no date restriction. The number of included studies in the reviews ranged from 10 to 131. Six reviews included over 50 studies, 7 reviews included between 30 and 49 studies, and 6 reviews included fewer than 29 studies. Of the 19 systematic reviews, there were 11 meta-analyses and 8 summative syntheses, whereby a nonstatistical and narrative synthesis describing the findings was conducted. Details of included reviews are presented in Supplementary Table S2. The review characteristics are summarized below in Table 1.

**Table 1.** Summary of Review Characteristics

Characteristics of reviews (*n* = 19)	No. of reviews	References
Date of publication		
2010	2	^34,63^
2014	3	^23,58,65^
2015	4	^50,52,53,60^
2016	2	^55,59^
2017	3	^54,56,66^
2018	5	^51,57,61,62,64^
Geographical location		
No limits	15	^23,34,50–54,57–63,65^
High-income or OECD countries	4	^55,56,64,66^
Study design filter		
No limits	11	^34,50–52,54,55,57,59–61,65^
Cross-sectional only	4	^23,53,58,62^
Cross-sectional and longitudinal	4	^56,63,64,66^
Age		
No limits	1	^54^
CYP	18	^23,34,50–53,55–66^
Other participant characteristics		
No targeting	7	^52–22,58,60,61^
Healthy CYP	10	^23,34,50,56,57,59,62–65^
LGBTQ	1	^51^
Victims of bullying	1	^66^
Search start date		
Not stated	6	^53–55,57,59,66^
1910 or no date restriction	6	^23,50,51,58,63,65^
1990–2000	5	^52,56,60–62^
2001–2010	2	^34,64^
No. of included studies		
fewer than 29	6	^34,50,51,53,57,61^
30–50	7	^56,58,60,62–65^
More than 50	6	^23,52,54,55,59,66^
Types of synthesis		
Summative synthesis	8	^34,50–53,60,63,64^
Meta-analysis	11	^23,54–59,61,62,65,66^

CYP, children and young people; LGBTQ, lesbian, gay, bisexual, transsexual, and questioning; OECD, Organisation for Economic Co-operation and Development.

### Outcomes measured

A summary of the outcomes reported is presented below in Table 2. All of the reviews reported outcomes associated with cyberbullying, which we grouped into three categories: mental health outcomes, psychosocial outcomes, and contextual/moderating factors. The most commonly reported mental health outcomes were measures of depression (*n* = 14), anxiety (*n* = 10), hostility/aggression (*n* = 6), and suicidality (*n* = 11). Self-harm, often linked to suicidality in the literature, was also reported in four reviews. Other outcomes included in single reviews were loneliness and attention-deficit hyperactivity disorder (ADHD)/hyperactivity.

**Table 2.** Summary of Outcome Measures

Outcomes associated with cyberbullying	No. of reviews (*n* = 19)	References
Mental health outcomes		
Depression	14	^23,34,50–57,60,63,64,66^
Suicidality	11	^23,50,51,53,55,56,58,60,62,63,65^
Anxiety	10	^23,34,52,53,55–57,60,64,66^
Hostility and aggression	6	^23,51,53,55,60,64^
Substance misuse/use	6	^23,50,52,53,55,60^
Self-harm	4	^55,56,60,61^
ADHD symptoms/self-regulation	1	^64^
Psychosocial outcomes		
Self-esteem	9	^23,51–56,60,64^
Peer problems/bullying	10	^51–57,59–61^
Substance misuse/use	6	^23,51–53,55,60^
Stress/distress	6	^23,34,51,53,55,60^
Well-being/life satisfaction	3	^23,55,56^
Social support/social skills	2	^54,64^
Loneliness	1	^23^
Moderating factors		
Demographics	12	^23,34,51–53,56,59–61,63,64,66^
School factors	9	^23,34,51–54,59,60,64^
Parenting/family factors	8	^23,34,51,52,54,59,60,64^
Personality traits/temperament	8	^23,34,50–53,59,60^

ADHD, attention-deficit hyperactivity disorder.

With respect to psychosocial outcomes, self-esteem (*n* = 9) and peer relationship problems (*n* = 10) were most commonly reported, followed by substance misuse (*n* = 6) and stress/distress (*n* = 6). Life satisfaction and social support featured in three and two reviews, respectively. Other psychosocial outcomes such as anger, fear, isolation, and loss of confidence also featured, but with less prominence.

### Moderator analysis

The reviews also cited evidence on factors potentially moderating the impact of cyberbullying, generally reported in terms of demographics (age and gender) (*n* = 12), contextual and school factors (*n* = 17), and individual factors (*n* = 8). Males were reported to be associated with higher levels of cyberbullying perpetration^52,59,64^ and females significantly more likely to be cybervictims,^61^ so were LGBTQ.^51^ A negative school climate, poor family communication and peer rejection,^23,34,52,54,59,60,64^ socioeconomic status,^52,64^ and traditional bullying^23,52,57,59^ had the potential to influence the strength of this likelihood. Personal traits and temperament such as hyperactivity, aggression, antisocial behaviors, and moral disengagement were also linked to cyberbullying perpetuation.^23,34,50–53,59^

### Risk of bias/quality of the reviews

The quality of the reviews varied. Only three were classified to be of high quality. Of the remaining 16 reviews, six were judged to be of unclear quality and 10 of low quality. When exploring risk of bias within individual domains we found the following:

Low risk of bias was identified in four domains in reviews, which

○ reported an explicit aim/research question and inclusion criteria (*n* = 19)
○ employed a fully comprehensive search strategy (*n* = 13)
○ provided a full description of the included studies (*n* = 11)
○ reported conflicts of interest (*n* = 11)

Unclear risk of bias was identified in two domains in reviews, which did not

○ report conflict of interests (*n* = 4)
○ conduct duplicate data extraction (*n* = 9)

High risk of bias was identified in the remaining seven domains in reviews, which failed to

○ refer to an existing protocol (*n* = 17)
○ provide a rationale for study design eligibility criteria (*n* = 12)
○ conduct duplicate screening (*n* = 12)
○ report their reasons for excluding studies (*n* = 12)
○ conduct any form of critical appraisal (*n* = 15)
○ provide funding details of included studies (*n* = 19)
○ reflect on the quality of the evidence base when interpreting the findings (*n* = 12)

**FIG. 2.**
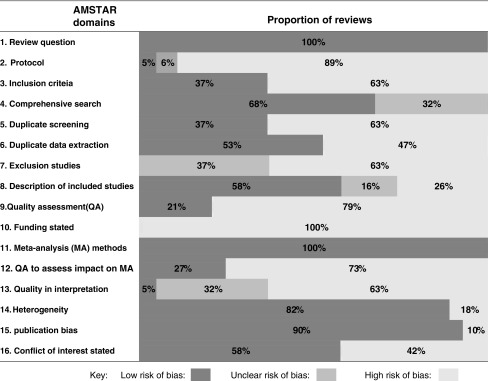
Summary of risk of bias for each AMSTAR domain.

We assessed the quality of the 11 meta-analyses according to AMSTAR criteria. Nine of the 11 reviews explored sources of heterogeneity and publication bias and 11 examined risk of bias as a source of heterogeneity. However, eight reviews did not adequately assess the potential impact of risk of bias of individual studies on the result of the meta-analysis. Risk of bias was only discussed partially in the interpretation of the findings. Summaries of quality assessment of reviews are presented in Table 3 and Figure 2, respectively.

**Table 3.** Quality Assessment by High-, Unclear, and Low-Quality Weighting

Author (year)	AMSTAR domains reported																Overall rating
	1. Review question stated	2. Protocol?	3. Inclusion criteria	4. Comprehensive search strategy?	5. Duplicate screening?	6. Duplicate data Extraction?	7.Exclusions?	8. Included studies described?	9. QA of studies?	10. Funding stated?	11. Appropriate meta-analysis methods?	12. QA used to assess impact on meta-analysis?	13. Quality in interpretation	14. Heterogeneity explored?	15. Publication bias	16. Conflict of interest stated?	
Bottino et al. (2015)^53^	+	−	+	±	+	−	±	+	+	−	N/A	N/A	−	N/A	N/A	−	High
Fisher et al. (2016)^55^	+	−	+	+	+	+	±	±	+	−	+	+	+	+	+	+	High
John et al. (2018)^62^	+	+	−	+	+	+	±	+	+	−	+	+	±	+	+	+	High
Abreu and Kenny (2018)^51^	+	−	+	+	+	+	−	+	−	−	N/A	N/A	–	N/A	N/A	+	Unclear
Chen et al. (2017)^54^	+	−	−	±	−	+	±	±	−	−	+	−	−	+	−	−	Unclear
Guo (2016)^59^	+	−	−	+	−	+	±	+	−	−	+	−	±	+	+	−	Unclear
Hamm et al. (2015)^60^	+	−	−	+	+	+	±	±	+	−	N/A	N/A	–	N/A	N/A	+	Unclear
Heerde and Hemphill (2018)^61^	+	−	+	±	+	−	−	+	−	−	+	−	−	+	+	−	Unclear
van Geel et al. (2014)^65^	+	−	−	+	−	+	±	+	−	−	+	−	−	−	+	+	Unclear
Aboujaoude et al. (2015)^50^	+	−	−	+	−	−	−	+	−	−	N/A	N/A	−	N/A	N/A	+	Low
Baldry et al. (2015)^52^	+	−	−	±	−	−	−	−	−	−	N/A	N/A	–	N/A	N/A	+	Low
Foody et al. (2017)^56^	+	±	+	+	−	−	−	+	−	−	+	−	±	−	+	+	Low
Gini and Espelage (2014)^58^	+	−	−	±	+	+	−	−	−	−	+	−	±	+	+	+	Low
Gini et al. (2018)^57^	+	−	−	+	−	+	−	+	−	−	+	−	−	+	+	−	Low
Klomek et al. (2010)^63^	+	−	+	+	−	−	−	−	−	−	N/A	N/A	±	N/A	N/A	+	Low
Kowalski et al. (2014)^23^	+	−	−	+	−	+	−	−	−	−	+	+	±	+	+	−	Low
Lee et al. (2018)^64^	+	−	+	+	−	−	−	+	−	−	N/A	N/A	−	N/A	N/A	−	Low
Tokunaga (2010)^34^	+	−	−	+	−	−	−	+	−	−	N/A	N/A	−	N/A	N/A	−	Low
Yuchang et al. (2017)^66^	+	−	−	±	−	−	−	−	−	−	+	−	−	+	+	+	Low

+, yes, low risk of bias; − , no, high risk of bias; ± , partial yes, unclear risk of bias; N/A, not applicable

QA, quality assessment.

## Discussion

As availability, accessibility, and functionality of Internet-enabled devises continue to develop, the potential for cyberbullying increases, along with its negative impact on CYP's mental health. The findings from our systematic map of reviews reflect the recent growth of interest and concerns in this area, with nearly three quarters of the reviews (14/19 [74 percent]) published after 2014. This shows a strong negative association between cyberbullying and mental health and psychosocial outcomes in CYP. The outcomes most commonly associated with cyberbullying were depression, suicidality, anxiety, hostility/aggression, substance misuse/use, self-harm, and ADHD/hyperactivity, in addition to low self-esteem, peer problems, stress/distress, loneliness, and life satisfaction. However, since none of the reviews provides longitudinal evidence, the extent to which mental health outcomes may be both the consequences of and precursors to cyberbullying remains difficult to establish.

### Comparison with the literature

Other evidence suggests that CYP are likely to experience mental health problems as a consequence of cyberbullying.^23,54^ Likewise, those with pre-existing mental health problems, such as depression, are also more likely than their peers to be bullied,^67,68^ suggesting the existence of a vicious circle, whereby psychosocial problems increase the risk of cyberbullying, which in turn exacerbates psychosocial problems.^69,70^ Nevertheless, lived experiences of CYP can attest to the harmful effect of cyberbullying on their mental health. While we did not identify any qualitative evidence synthesis of CYP's views of cyberbullying, primary qualitative investigations have suggested that adolescents perceive cyberbullying to be a potent strategy aimed to hurt girls, who experience lower self-esteem and feelings of depression, while boys tend to either act out by hitting back at the cyberbully using violence or take no offence.^71^ One primary qualitative study^72^ reported that adolescents considered public forms of cyberbullying on social networking sites (SNSs) to be worse than private exchanges. Not knowing the identity of the perpetuator intensified the impact, but the intensity was higher if they knew and were close to the perpetuator. The ability to orchestrate the removal of abusive messages posted onto SNS platforms also played a role in reducing the victims' stress. Bystander support from fellow SNS users could buffer victims against potential negative impact too.^72^ These qualitative data provide an important first step in the development and validation of an empirical framework for understanding the factors that moderate the adverse impact for adolescent cyberbullying victims. However, not all cyberbullying victims are negatively affected by their victimization.^29,73^ Young people considered adopting a personal coping strategy of general resilience, such as positivity, high self-esteem, and confidence, to be a protective factor against the distress caused by cyberbullying.^72^

The included reviews provided further insight into the extent to which the impact of cyberbullying can be moderated and mediated by demographic and social factors. Further exploration of the dynamic interplay of these factors can expand our understanding of the mechanism by which cyberbullying experiences and psychosocial outcomes are related. It helps to generate practical information in identifying effective intervention components,^74^ tailoring interventions for targeted populations. Given the high personal and societal costs in terms of short- and long-term consequences of cyberbullying, adopting different approaches to meet this challenge warrants additional research attention. It has been suggested that approaches in cyberbullying research has exclusively focused on the bully-victim dyad^75^ and that bystander support can be a resource in tackling cyberbullying. Based on the link between moral disengagement and aggressive behavior,^76^ innovative ideas have been developed to explore the role of electronic bystanders and the potential of moral educational efforts and assertive training to promote defending bystander behaviour and empower bystanders to become peer helpers in this context.^77,78^

### Gaps in evidence and implications for research

Cyberbullying is a relatively new concept. The absence of a universally accepted definition for cyberbullying^16^ is reflected in the evidence base, where there are also inconsistencies in the use of terminology, eligibility criteria (e.g., when subjects last experience cyberbullying: 3 or 6 months before study or lifetime), measurement tools, and internet modalities engaged (platforms such as social media, instant text messaging, chatrooms, PC cafés, e-mails, and/or other means), all of which are important methodological variables that could potentially contribute to the considerable variations in prevalence rates of cyberbullying in existing literature.^23,34,50^

There is a lack of evidence synthesis of longitudinal primary research on cyberbullying and mental health. While cross-sectional evidence, which measures outcomes at one point in time, can identify associations, longitudinal evidence is able to identify whether exposure (e.g., to cyberbullying) precedes any effect (e.g., on mental health), thereby indicating a causal relationship. Overall, the potential for new evidence synthesis hinges on the availability of longitudinal primary research that explores the following:

- the long-term consequences of cyberbullying on CYP's mental health;
- whether there is a dose–response relationship between cyberbullying and mental health;
- whether cyberbullying acts as either an antecedent and/or consequence of mental health and psychosocial well-being; and
- whether these temporal relationships are moderated by contextual factors and the mechanism of interaction across different modalities and CYP population groups.

A meta-synthesis of these reviews could also be used to inform future primary research on designing prevention programs, for example, by further exploring the risk factors that lead to cyberbullying perpetration and victimization.

None of the 19 reviews explored CYP's experiences of cyberbullying by conducting a qualitative evidence synthesis. It is unclear if this is due to a lack of primary research or lack of evidence at review level. Perceptions and experiences of cyberbullying are crucial to the understanding of the impact of cyberbullying as these data can inform clinicians working with CYP to identify and assess their mental health needs, a first step toward planning effective intervention.

With the exception of one review based on primary studies from Asia (South Korea),^64^ most primary research was conducted in North America, Europe, and Australia. There is some evidence that the prevalence of cyberbullying varied between different cultures and Asia has the highest level of cyberbullying,^79^ yet there is limited research from a cross-cultural perspective.^80^ The variation between diverse cultures, for example, between China and the West, is likely to influence adolescent cyberbullying behaviors and experiences. Future comparative studies taking a cross-cultural perspective could further our understanding of cyberbullying in this context.

The methodological quality of the systematic reviews varied considerably, with over two-thirds classified to be of unclear and low quality (16/19, see Table 3). The major shortcoming was the lack of quality assessment of the primary studies included and this aspect was not fully considered when interpreting the results of the summative synthesis and meta-analyses. Thus, future evidence synthesis would benefit from providing policy makers and practitioners a greater understanding of the trustworthiness of the evidence base by conducting a thorough critical appraisal of the studies included in reviews.

Building on existing evidence, future research should also consider studies that examine the following: the mental health impact of cyberbullying on subsets of CYP populations (such as black minority ethnic group, LGBTQ, and CYP with disabilities); how cyberbullying behaviors, experiences, and impact vary when using different internet modalities; the interaction between the different moderating factors in identifying populations amenable for resilience building, together with investigating whether and which resilience factors (such as confidence, self-efficacy, and social competency) could mitigate the emotional damage to young cyberbullying victims.

### Strengths and limitations

To the best of our knowledge, this is the first comprehensive systematic map summarizing review-level evidence on cyberbullying and mental health and psychosocial outcomes in CYP. In conducting an extensive and comprehensive search that was not limited by a predefined set of mental health and psychosocial outcomes, we were able to explore the breadth of review literature undertaken in this field. This systematic map provides a descriptive overview of review-level research activity, not a meta-synthesis of findings. Unlike most maps, we critically assessed the included reviews for their methodological quality, enabling us to make judgments about the quality of the evidence base. However, we have not examined the size of the primary evidence base for each outcome. We have judged the quality of the reviews, but we do not know the quality of the primary studies within each of the reviews, which would require further in-depth synthesis.

The search was limited to the last ten years, to coincide with the advent of web 2.0 technologies (the introduction of the Smartphone) and to reviews indexed in English-language databases and reported in the English language. Thus, further evidence syntheses may have been conducted in older reviews and other languages. The extent to which the systematic reviews shared the same primary studies remains unknown, and “double counting” is highly likely due to some degree of overlap between the primary studies within the 19 reviews included in this study.

Evidence from this map contributed to the understanding of the impact of screen-based activities in general on CYP's mental health and psychosocial well-being and informs the development of a national digital and social media policy.^41^ The greater availability of this type of knowledge is vital to support policymakers, parents, CYP, and the wider community make informed choices about how they engage with online activities.

## Conclusions

As the Internet enters its fourth decade, over 55 percent of the world's population now has access to the Internet.^81^ The permanency of the Internet seems inevitable, with its increased encroachment into private aspects of our lives.^82,83^ In a digital future of hyperconnectivity bringing us ever closer to one another online, the ubiquity and the insidious nature of cyberbullying will grow, requiring a comprehensive strategic approach to safeguard vulnerable users and restore citizens' confidence in technology. This systematic map consolidates available review-level evidence, which confirms the strong negative association between cyberbullying and mental health and psychosocial outcomes in CYP. We identify the research gaps in longitudinal and qualitative evidence synthesis. Closer examination of the potential of moderating factors influencing cyberbullying behaviors would merit serious consideration in future research to advance our understanding and inform the development of tailored programs of intervention to mitigate the negative impact of this phenomenon.

## Disclaimer

The views expressed in this publication are those of the author(s) and not necessarily those of the NHS, the National Institute for Health Research or the Department of Health and Social Care.

## Supplementary Material

Supplemental data

Supplemental data

Supplemental data
