# Supplementary material for: Cyberbullying and Children and Young People's Mental Health: A Systematic Map of Systematic Reviews
Source: Cyberpsychol Behav Soc Netw. 2020 Feb 5;23(2):72–82. doi: 10.1089/cyber.2019.0370 (PMC7044782; doi:10.1089/cyber.2019.0370)
Supplement: Supplemental data [file Supp_TableS1.pdf]

Supplementary Data

Supplementary Table S1. Example of Coding Tool

Domains		Criteria/guidance: Code	Codes e.g.
Review focus and aims	Publication year	The year the review was published	2010, 2011, 2012, etc.
	Focus	Cyberbullying	
	Aims of included reviews	Key aims of the review	Associations between screen-based activity and MH outcomes<br>Longitudinal associations (e.g., risk factors and consequences of cyberbullying)<br>CYP's view/experiences
Review scope[a]	Geographical location	Any geographical limit placed on inclusion of studies	High-income countries only<br>No geographical limits placed
	Population: age	Age range reviews focused on	CYP 0–25 years only<br>No age limit placed
	Population: other targeting	Any focus on other CYP population characteristics of interest	CYP with mental health issues<br>No population targeting
	Study design	Study design inclusion criteria	Longitudinal studies only<br>Quantitative studies, etc.
	Date range searched	The date range of search reported in methods	Provide year to year as stated in the reviews<br>Not reported
	No. of included articles	The number of articles that passed inclusion screening	N = articles included<br>Not reported
	Type of synthesis	Approach taken to combine and analyze the data	Summative synthesis[b];<br>Meta-analysis<br>Qualitative evidence synthesis
Outcomes and factors	Associations between cyberbullying and outcomes	Code outcomes and factors	Mental health outcomes: (e.g., depression, anxiety, and self-esteem)
	Longitudinal risk factors of cyberbullying		Psychosocial outcomes (e.g., life satisfaction and social connectedness)
	Longitudinal consequences of cyberbullying		Factors (e.g., personality traits and demographics)

[a]Based on the review eligibility criteria, not the description of included primary studies.
[b]Where summary statements made about the quantity of evidence, such as 'two studies found an association between screen time and anxiety, but which do not conduct meta-analysis.
CYP, children and young people; MH, mental health.
