# Supplementary material for: Cyberbullying and Children and Young People's Mental Health: A Systematic Map of Systematic Reviews
Source: Cyberpsychol Behav Soc Netw. 2020 Feb 5;23(2):72–82. doi: 10.1089/cyber.2019.0370 (PMC7044782; doi:10.1089/cyber.2019.0370)
Supplement: Supplemental data [file Supp_TableS2.pdf]

Supplementary Table S2. Characteristics of Included Reviews

Author (year)	*Review characteristics*	*Date range searched*	*Included articles*	*Type of synthesis*	*Type of data synthesized*	*Mental health and psychosocial outcomes*	*Other variables*
Aboujaoude et al. (2015)[S1]	Geographical location: no filter<br>Population: CYP and young adults only<br>Study designs: no filter<br>Other targeting: healthy CYP	No date restriction	26	Summative synthesis	Associations (26 cross-sectional)	Depression<br>Suicidality<br>Substance misuse<br>Stress/distress	Personality traits/temperament
Abreu and Kenny (2018)[S2]	Geographical location: no filter<br>Population: CYP and young adults only<br>Study designs: no filter<br>Other targeting: LGBTQ+ CYP	August 2016–March 2017	27	Summative synthesis	Associations (mixed: cross-sectional, mixed methods, and qualitative studies)	Depression<br>Hostility/aggression<br>Suicidality<br>Self-esteem<br>Peer problems/bullying	Demographics<br>Parenting/family factors<br>School factors<br>Personality traits/temperament
Baldry et al. (2015)[S3]	Geographical location: no filter<br>Population: CYP and young adults only<br>Study designs: no filter<br>Other targeting: none	2000–February 2015	53	Summative synthesis	Associations (mixed: cross-sectional, mixed methods, and qualitative studies)	Depression<br>Anxiety<br>Substance misuse<br>Self-esteem<br>Peer problems/bullying	Demographics<br>Parenting/family factors<br>School factors<br>Personality traits/temperament
Bottino et al. (2015)[S4]	Geographical location: no filter<br>Population: CYP and young adults only<br>Study designs: cross-sectional<br>Other targeting: none	Not stated	10	Summative synthesis	Associations (10 cross-sectional)	Depression<br>Anxiety<br>Hostility/aggression<br>Suicidality<br>Self-harm<br>Substance misuse<br>Self-esteem<br>Stress/distress<br>Peer problems/bullying	Demographics<br>School factors<br>Personality traits/temperament
Chen et al. (2017)[S5]	Geographical location: no filter<br>Population: no filter<br>Study designs: quantitative<br>Other targeting: none	Not stated	81	Meta-analysis	Associations (81cross-sectional)	Depression<br>Self-esteem	Parenting/family factors<br>School factors<br>Social support/social skills

(*continued*)

Author (year)	*Review characteristics*	*Date range searched*	*Included articles*	*Type of synthesis*	*Type of data synthesized*	*Mental health and psychosocial outcomes*	*Other variables*
Fisher et al. (2016)[S6]	Geographical location: US<br>Population: CYP and young adults only<br>Study designs: quantitative<br>Other targeting: none	Not stated	55	Meta-analysis	Associations (55 cross-sectional)	Depression<br>Anxiety<br>Hostility/aggression<br>Suicidality<br>Self-harm<br>Substance misuse<br>Stress<br>Self-esteem<br>Well-being/life satisfaction<br>Peer problems/bullying	
Foody et al. (2017)[S7]	Geographical location: Northern or the Republic of Ireland<br>Population: CYP and young adults only<br>Study designs: cross-sectional and longitudinal<br>Other targeting: healthy CYP	January 1997–April 2016	39	Meta-analysis	Associations (mixed: cross-sectional, mixed methods, and qualitative studies)	Depression<br>Anxiety<br>Suicidality<br>Self-harm<br>Self-esteem<br>Well-being/life satisfaction<br>Peer problems/bullying	Demographics
Gini (2018)[S8]	Geographical location: no filter<br>Population: CYP and young adults only<br>Study designs: no filter<br>Other targeting: healthy CYP	2009–2015	20	Meta-analysis	Associations (20 cross-sectional)	Depression<br>Anxiety<br>Peer problems/bullying	
Gini and Espelage (2014)[S9]	Geographical location: no filter<br>Population: CYP and young adults only<br>Study designs: no filter<br>Other targeting: none	1910–2013	34	Meta-analysis	Associations (34 cross-sectional)	Suicidality	
Guo et al. (2016)[S10]	Geographical location: no filter<br>Population: CYP and young adults only<br>Study designs: quantitative<br>Other targeting: healthy CYP	Not stated	77	Meta-analysis	Associations (mixed: cross-sectional, mixed methods, and qualitative studies)	Internalizing problems<br>Externalizing problems	Demographics<br>Parenting/family factors<br>School factors<br>Personality traits/temperament

(*continued*)

Author (year)	*Review characteristics*	*Date range searched*	*Included articles*	*Type of synthesis*	*Type of data synthesized*	*Mental health and psychosocial outcomes*	*Other variables*
Hamm et al. (2015)[S11]	Geographical location: no filter<br>Population: CYP and young adults only<br>Study designs: no filter<br>Other targeting: none	January 2000–June 2014	34	Summative synthesis	Associations (mixed: cross-sectional, mixed methods, and qualitative studies)	Depression<br>Anxiety<br>Hostility/aggression<br>Suicidality<br>Self-harm<br>Substance misuse<br>Self-esteem<br>Stress/distress<br>Peer problems/bullying<br>Other mental health	Demographics<br>Parenting/family factors<br>School factors<br>Personality traits/temperament
Heerde and Hemphill (2018)[S12]	Geographical location: no filter<br>Population: CYP and young adults only<br>Study designs: no filter<br>Other targeting: healthy CYP	1990–2018	27	Meta-analysis	Associations (mixed: cross-sectional, mixed methods, and qualitative studies)	Self-harm<br>peer problems/bullying	Demographics
John et al. (2018)[S13]	Geographical location: no filter<br>Population: CYP and young adults only<br>Study designs: cross-sectional<br>Other targeting: healthy CYP	January 1996–February 2017	33	Meta-analysis	Associations (mixed: cross-sectional, mixed methods, and qualitative studies)	Suicidality<br>Self-harm	
Klomek et al. (2010)[S14]	Geographical location: no filter<br>Population: CYP and young adults only<br>Study designs: cross-sectional and longitudinal<br>Other targeting: healthy CYP	No date restriction	31	Summative synthesis	Associations (mixed: cross-sectional, mixed methods, and qualitative studies)	Suicidality<br>Depression	
Kowalski et al. (2014)[S15]	Geographical location: no filter<br>Population: CYP and young adults only<br>Study designs: cross-sectional<br>Other targeting: healthy CYP	No date restriction	131	Meta-analysis	Associations (131 cross-sectional)	Depression<br>Anxiety<br>Loneliness<br>Hostility/aggression<br>Suicidality<br>Substance misuse<br>Self-esteem<br>Well-being/life satisfaction<br>Stress/distress<br>Other mental health	Demographics<br>Parenting/family factors<br>School factors<br>Personality traits/temperament

(*continued*)

Supplementary Table S2. (Continued)

Author (year)	*Review characteristics*	*Date range searched*	*Included articles*	*Type of synthesis*	*Type of data synthesized*	*Mental health and psychosocial outcomes*	*Other variables*
Lee et al. (2018)[S16]	Geographical location: South Korea<br>Population: CYP and young adults only<br>Study designs: quantitative, qualitative, and mixed methods<br>Other targeting: healthy CYP	2005–2015	38	Summative synthesis	Associations (mixed: cross-sectional, mixed methods, and qualitative studies)	Depression<br>Anxiety<br>Hostility/aggression<br>ADHD symptoms/ self-regulation<br>Self-esteem	Demographics<br>Parenting/family factors<br>School factors<br>Social support/social skills<br>Internet use
Tokunaga (2010)[S17]	Geographical location: no filter<br>Population: CYP and young adults only<br>Study designs: no filter<br>Other targeting: healthy CYP	Publication prior to June 2009	25	Summative synthesis	Associations (25 cross-sectional)	Depression<br>Anxiety<br>Self-esteem<br>Stress/distress	Demographics<br>Parenting/family factors<br>School factors<br>Personality traits/temperament
Van Geel et al. (2014)[S18]	Geographical location: no filter<br>Population: CYP and young adults only<br>Study designs: no filter<br>Other targeting: healthy CYP	January 1910–January 2013	36	Meta-analysis	Associations (36 cross-sectional)	Suicidality	
Yuchang et al. (2017)[S19]	Geographical location: North America, Europe Australia, and China<br>Population: CYP and young adults only<br>Study designs: quantitative<br>Other targeting: victims of bullying	Not stated	56	Meta-analysis	Associations (mixed: cross-sectional, mixed methods, and qualitative studies)	Depression<br>Anxiety	Demographics

ADHD, attention-deficit hyperactivity disorder.

Supplementary References

S1. Aboujaoude E, Savage MW, Starcevic V, Salame WO. Cyberbullying: review of an old problem gone viral. Journal of Adolescent Health 2015; 57:10–18.

S2. Abreu RL, Kenny MC. Cyberbullying and LGBTQ youth: a systematic literature review and recommendations for prevention and intervention. Journal of Child and Adolescent Trauma 2018; 11:81–97.

S3. Baldry Anna C, Farrington David P, Sorrentino A. "Am I at risk of cyberbullying"? A narrative review and conceptual framework for research on risk of cyberbullying and cybervictimization: the risk and needs assessment approach. Aggression and Violent Behavior 2015; 23:36–51.

S4. Bottino SM, Bottino CM, Regina CG, et al. Cyberbullying and adolescent mental health: systematic review. Cadernos de Saúde Publica 2015; 31:463–475.

S5. Chen L, Ho S, Lwin M. A meta-analysis of factors predicting cyberbullying perpetration and victimization: from the social cognitive and media effects approach. New Media & Society 2017; 19:1194–1213.

S6. Fisher B, Gardell J, Teurbe-Tolon A. Peer cybervictimization among adolescents and the associated internalizing and externalizing problems: a meta-analysis. Journal of Youth and Adolescence 2016; 45:1727–1743.

S7. Foody M, Samara M, O'Higgins Norman J. Bullying and cyberbullying studies in the school-aged population on the island of Ireland: a meta-analysis. British Journal of Educational Psychology 2017; 87:535–557.

S8. Gini G, Card NA, Pozzoli T. A meta-analysis of the differential relations of traditional and cyber-victimization with internalizing problems. Aggressive Behavior 2018; 44:185–198.

S9. Gini G, Espelage DL. Peer victimization, cyberbullying, and suicide risk in children and adolescents. JAMA 2014; 312:545–546.

S10. Guo SY. A meta-analysis of the predictors of cyberbullying perpetration and victimization. Psychology in the Schools 2016; 53:432–453.

S11. Hamm MP, Newton AS, Chisholm A, et al. Prevalence and effect of cyberbullying on children and young people: a scoping review of social media studies. JAMA Pediatrics 2015; 169:770–777.

S12. Heerde JA, Hemphill SA. Are bullying perpetration and victimization associated with adolescent deliberate self-harm? A meta-analysis. Archives of Suicide Research 2013; 23:353–381.

S13. John A, Glendenning AC, Marchant A, et al. Self-harm, suicidal behaviours, and cyberbullying in children and young people: systematic review. Journal of Medical Internet Research 2018; 20:e129.

S14. Klomek AB, Sourander A, Gould M. The association of suicide and bullying in childhood to young adulthood: a review of cross-sectional and longitudinal research findings. The Canadian Journal of Psychiatry/La Revue canadienne de psychiatrie 2010; 55:282–288.

S15. Kowalski RM, Giumetti GW, Schroeder AN, Lattanner MR. Bullying in the digital age: a critical review and meta-analysis of cyberbullying research among youth. Psychological Bulletin 2014; 140:1073–1137.

S16. Lee JM, Hong JS, Yoon J, et al. Correlates of adolescent cyberbullying in South Korea in multiple contexts: a review of the literature and implications for research and school practice. Deviant Behavior 2018; 39:293–308.

S17. Tokunaga RS. Following you home from school: a critical review and synthesis of research on cyberbullying victimization. Computers in Human Behavior 2010; 26:277–287.

S18. van Geel M, Vedder P, Tanilon J. Relationship between peer victimization, cyberbullying, and suicide in children and adolescents: a meta-analysis. JAMA Pediatrics 2014; 168:435–442.

S19. Yuchang J, Junyi L, Junxiu A, et al. The differential victimization associated with depression and anxiety in cross-cultural perspective: a meta-analysis. Trauma Violence & Abuse 2017; 20:560–573.
