# Supplementary material for: Cyberbullying and Children and Young People's Mental Health: A Systematic Map of Systematic Reviews
Source: Cyberpsychol Behav Soc Netw. 2020 Feb 5;23(2):72–82. doi: 10.1089/cyber.2019.0370 (PMC7044782; doi:10.1089/cyber.2019.0370)
Supplement: Supplemental data [file Supp_TableS3-S4.pdf]

Supplementary Table S3. AMSTAR 2 Adapted Coding Tool

Question	*Guidance*	*Answer*
1. Review question and inclusion criteria: *Did the research questions and inclusion criteria for the review include the components of PICO?*	*Yes: There is an explicit aim/research question and inclusion criteria, focusing on the following:*<br>*P = Population*<br>*I = Intervention, Prognostic Factor, Exposure (can be N/A)*<br>*C = Comparison (can be None or placebo.)*<br>*O = Outcome: IV (independent variable)+DV (dependent variable)*<br>*No*	Yes<br>No
2. Protocol *Did the report of the review contain an explicit statement that the review methods were established before the conduct of the review and did the report justify any significant deviation from the protocol?*	*Yes: The authors state that they had a written protocol or guide that included ALL the following:*<br>*review question(s)*<br>*a search strategy*<br>*inclusion/exclusion criteria*<br>*a risk of bias assessment*<br>*the protocol should be registered and should also have specified the following:*<br>*a meta-analysis/synthesis plan, if appropriate, and*<br>*a plan for investigating causes of heterogeneity*<br>*justification for any deviation*<br>*For Partial Yes:*<br>*The authors state that they had a written protocol or guide that included ALL the following:*<br>*review question(s)*<br>*a search strategy*<br>*inclusion/exclusion criteria*<br>*a risk of bias assessment*<br>*No*	Yes<br>Partial Yes<br>No
3. Study designs included *Did the review authors explain their selection of the study designs for inclusion in the review?*	*Yes: the review should satisfy ONE of the following:*<br>*Explanation for including observational studies (including longitudinal and cross-sectional)*<br>*No*	Yes<br>No
4. Search strategy *Did the review authors use a comprehensive literature search strategy?*	*Yes:*<br>*searched at least 2 databases (relevant to research question)*<br>*provided key word and/or search strategy*<br>*justified publication restrictions (*e.g.*, language)*<br>*searched ref lists of included studies*<br>*searched gray literature*<br>*conducted search within 24mths of completion of review*<br>*Partial Yes*<br>*All of the following:*<br>*searched at least 2 relevant databases*<br>*provided keyword and/or search strategy*<br>*No*	Yes<br>Partial Yes<br>No
5. Double screening *Did the review authors perform study selection in duplicate?*	*Yes: either ONE of the following:*<br>*at least two reviewers independently agreed on selection of eligible studies and achieved consensus on which studies to include OR two reviewers selected a sample of eligible studies and achieved good agreement (at least 80 percent), with the remainder selected by one reviewer.*<br>*No*	Yes<br>No
6. Double data extraction? *Did the review authors perform data extraction in duplicate?*	*Yes: For Yes, either ONE of the following:*<br>*at least two reviewers achieved consensus on which data to extract from included studies*<br>*OR two reviewers extracted data from a sample of eligible studies and achieved good agreement (at least 80 percent), with the remainder extracted by one reviewer.*<br>*No*	Yes<br>Partial Yes<br>No

(*continued*)

Supplementary Table S3. (Continued)

Question	*Guidance*	*Answer*
7. Excludes reported<br>*Did the review authors provide a list of excluded studies and justify the exclusions?*	*Yes: For yes provided a list of all potentially relevant studies that were read in full-text form, but excluded from the review not enough to report numbers and reasons. Need references too.*<br>*Partial Yes: reports numbers with reasons, but no references*<br>*No*	
8. Description of studies<br>*Did the review authors describe the included studies in adequate detail? [what is adequate]*	*Yes:*<br>*described population in detail*<br>*described research designs*<br>*described IV and DV/outcome in detail*<br>*described study's setting*<br>*timeframe for followup*<br>*described interventions [if relevant]*<br>*described comparators [if relevant]*<br>*Partial Yes*:<br>*described populations*<br>*described IV and DV/outcomes*<br>*described research designs*<br>*described interventions [if relevant]*<br>*described comparators [if relevant]*<br>*No*	Yes<br>Partial Yes<br>No
9. Quality appraisal<br>*Did the review authors' quality appraise the included studies?*	*Yes: must also have assessed risk of bias:*<br>*from confounding, and*<br>*from selection bias*<br>*methods used to ascertain IV and DV*<br>*selection of the reported result from among multiple measurements or analyses of a specified outcome [only if multiple measures,* e.g., *in longitudinal research]*<br>*Partial Yes*<br>*For Partial Yes, must have assessed RoB:*<br>*from confounding, and*<br>*from selection bias review?*<br>*No*	Yes<br>Partial Yes<br>No
10. Funding for included studies<br>*Did the review authors report on the sources of funding for the studies included in the review?*	*Yes: must have reported on the sources of funding for individual studies included in the review. Note: Reporting that the reviewers looked for this information, but it was not reported by study authors also qualify.*<br>*No*	Yes<br>No
11. Meta-analysis<br>*If meta-analysis was performed, did the review authors use appropriate methods for statistical combination of results?*	*For Yes (for NRSI):*<br>*The authors justified combining the data in a meta-analysis*<br>*AND they used an appropriate weighted technique to combine study results, adjusting for heterogeneity if present*<br>*AND they statistically combined effect estimates from NRSI that were adjusted for confounding, rather than combining raw data, or justified combining raw data when adjusted effect estimates were not available*<br>*AND they reported separate summary estimates for RCTs and NRSI separately when both were included in the review*<br>*No*<br>*N/A* (no MA conducted)	Yes<br>No<br>N/A
12. Quality in meta-analysis<br>*If meta-analysis was performed, did the review authors assess the potential impact of QUALITY [originally RoB] in individual studies on the results of the meta-analysis or other evidence synthesis?*	*Yes: the authors performed analyses to investigate possible impact of Risk of Bias on findings*<br>*No*<br>*N/A* (no MA conducted)	

(*continued*)

Supplementary Table S3. (Continued)

Question	*Guidance*	*Answer*
13. Quality in interpretation<br>*Did the review authors account for quality appraisal in individual studies when interpreting/discussing the results of the review?*	*Yes: the review provided a discussion of the likely impact of RoB on the results [not possible if RoB is not discussed in context of included studies]*<br>*Partial Yes: the review provided a discussion of the likely impact of bias* e.g., *in terms of study designs used*<br>*No*	Yes<br>Partial Yes<br>No
14. Heterogeneity<br>*Did the review authors provide a satisfactory explanation for, and discussion of, any heterogeneity observed in the results of the review?*	*Yes: There was no significant heterogeneity in the results OR if heterogeneity was present, the authors performed an investigation of sources of any heterogeneity in the results and discussed the impact of this on the results of the review*<br>*No*<br>*N/A* (no MA conducted)	
15. Publication bias<br>*If they performed quantitative synthesis [*i.e., *pooled results rather than summative], did the review authors carry out an adequate investigation of publication bias (small study bias) and discuss its likely impact on the results of the review?*	*Yes: performed graphical or statistical tests for publication bias and discussed the likelihood and magnitude of impact of publication bias*<br>*No*<br>*N/A* (no MA conducted)	Yes<br>No<br>N/A
16. Conflict/review funding<br>*Did the review authors report any potential sources of conflict of interest, including any funding they received for conducting the review*	*Yes: The authors reported no competing interests OR The authors described their funding sources and how they managed potential conflicts of interest*<br>*No*	

MA, meta-analysis; NRSI, non-randomized studies of interventions; RoB, risk of bias; RCTs, randomized controlled trials.

Supplementary Table S4. Categorization of Review Quality by Risk of Bias

Risk of bias	*Domains: yes/partial yes answer*
Low (high quality)	Review question and inclusion criteria: *did the review authors report an explicit aim/research question and PICO (Population, Intervention, Control, Outcomes) inclusion criteria?*<br>Search strategy: *Did the review authors use a comprehensive literature search strategy?*<br>Duplicate screening: *Did the review authors perform study selection in duplicate?*<br>Excludes reported: *Did the review authors provide a list of excluded studies and justify the exclusions?*<br>Description of studies: *Did the review authors describe the included studies in adequate detail?*<br>Quality appraisal: *Did the review authors' quality appraise the included studies?*
Unclear	Review question and inclusion criteria: *did the review authors report an explicit aim/research question and PICO (Population, Intervention, Control, Outcomes) inclusion criteria?*<br>Search strategy: *Did the review authors use a comprehensive literature search strategy?*<br>Duplicate screening: *Did the review authors perform study selection in duplicate?*<br>Excludes reported: *Did the review authors provide a list of excluded studies and justify the exclusions?*<br>Description of studies: *Did the review authors describe the included studies in adequate detail?*
High (low quality)	*Failed to meet at least one of these criteria.*
